# Hematological and plasma biochemical parameters in a wild population of *Naja naja* (Linnaeus, 1758) in Sri Lanka

**DOI:** 10.1186/s40409-017-0098-7

**Published:** 2017-02-13

**Authors:** Duminda S. B. Dissanayake, Lasanthika D. Thewarage, Rathnayake M. P. Manel Rathnayake, Senanayake A. M. Kularatne, Jamburagoda G. Shirani Ranasinghe, Rajapakse P. V. Jayantha Rajapakse

**Affiliations:** 1 Department of Veterinary Pathobiology, Faculty of Veterinary Medicine and Animal Science, University of Peradeniya, Peradeniya, Sri Lanka; 2 Department of Pathology, Faculty of Medicine, University of Peradeniya, Peradeniya, Sri Lanka; 3 Department of Medicine, Faculty of Medicine, University of Peradeniya, Peradeniya, Sri Lanka; 4 Department of Biochemistry, Faculty of Medicine, University of Peradeniya, Peradeniya, Sri Lanka

**Keywords:** Hematology, Blood cells, Plasma biochemistry, *Naja naja*

## Abstract

**Background:**

Hematological studies of any animal species comprise an important diagnostic method in veterinary medicine and an essential tool for the conservation of species. In Sri Lanka, this essential technique has been ignored in studies of many species including reptiles. The aim of the present work was to establish a reference range of hematological values and morphological characterization of wild spectacled cobras (*Naja naja*) in Sri Lanka in order to provide a diagnostic tool in the assessment of health condition in reptiles and to diagnose diseases in wild populations.

**Methods:**

Blood samples were collected from the ventral caudal vein of 30 wild-caught *Naja naja* (18 males and 12 females). Hematological analyses were performed using manual standard methods.

**Results:**

Several hematological parameters were examined and their mean values were: red blood cell count 0.581 ± 0.035 × 10^6^/μL in males; 0.4950 ± 0.0408 × 10^6^/μL in females; white blood cell count 12.45 ± 1.32 × 10^3^/μL in males; 11.98 ± 1.62 × 10^3^/μL in females; PCV (%) in males was 30.11 ± 1.93 and in females was 23.41 ± 1.67; hemoglobin (g/dL) was 7.6 ± 0.89 in males and 6.62 ± 1.49 in females; plasma protein (g/dL) was 5.11 ± 0.75 in males and 3.25 ± 0.74 in females; whereas cholesterol (mg/mL) was 4.09 ± 0.12 in males and 3.78 ± 0.42 in females. There were no significant differences in hematological parameters between the two genders except for erythrocyte count, thrombocyte count, hematocrit, hemoglobin, plasma protein, percentage of azurophil and heterophil. Intracellular parasites were not found in any of the studied specimens.

**Conclusion:**

Hematological and plasma biochemical parameters indicated a difference between geographically isolated populations and some values were significantly different between the two genders. These hematological results provide a reference range for Sri Lankan population of adult *Naja naja*.

## Background

Sri Lanka is home to 90 species of inland snakes (including all terrestrial, fossorial and freshwater species, but not the true marine or estuarine forms) [1, 2]. Of these, six species are considered to be highly or deadly venomous: the spectacled cobra (*Naja naja*), common krait (*Bungarus caeruleus*), Ceylon krait (*Bungarus ceylonicus*), Russell’s viper (*Daboia russelii*), hump-nosed viper (*Hypnale* spp.), and the saw-scaled viper (*Echis carinatus*) [2]. Among them the spectacled cobra, *Naja naja* (Linnaeus, 1758), is the only species of cobra found in Sri Lanka which is also widely distributed throughout many Asian countries including India, Pakistan, Bangladesh and southern Nepal [3, 4]. During the early taxonomic studies, Sri Lankan cobra was described as a subspecies, *Naja naja naja*, but it was considered as synonymous with the species *N. naja* in the taxonomic revision carried out by Wüster and Thorpe in 1992 [3]. This medically important species inhabits all the peneplains and other habitats of Sri Lanka including forests, open fields and urban areas, from sea level up to around 1500 m.

Hematological parameters are widely used tools that aid in monitoring animal health, reproductive status, disease status and in differentiation of physiological processes [5–8]. In addition, studies on hematological parameters have been carried out to determine the systematic relationship among certain species [9, 10]

The history of hematological studies of reptiles dates back to 1842 [11]. Currently, there is growing interest on reptile hematology as an important tool in their conservation, trading and also for research based on clinical pathology of these animals [12–19]. Collectively, there is a considerable number of hematological and plasma biochemical research on a variety of snake species in other regions [20–28]. However, most of such studies were based only on blood cell count and morphology [13, 16, 17].

The aim of the present study was to investigate hematology and plasma biochemistry of *N. naja* in Sri Lanka in relation to the blood cell morphology. The current findings will stand as reference data for health assessment studies, epidemiology and conservation of this remarkable species in Sri Lanka. Furthermore, these findings will allow the understanding of population processes, ecological relationships, geographical variations and physiological conditions of this species.

## Methods

### Snakes

Thirty healthy adult snakes (18 males and 12 females) were collected from different geographical locations in Sri Lanka from April to December 2014. All captured snakes were kept separately in a temperature regulated fiberglass terrarium under the supervision of the Division of Parasitology, Department of Veterinary Pathobiology, Faculty of Veterinary Medicine and Animal Science, University of Peradeniya. Morphometric data was collected from the live specimens prior to the blood collection: snout to vent length (SVL), length from the tip of snout to posterior margin of anal plate; tail length (TAL), length from posterior margin of anal plate to tip of tail; and the sum of SVL and TAL was used to obtain total length (TOTL).

### Blood collection and smear preparations

Since a healthy reptile can tolerate an acute loss up to 10% of the total blood volume about 1.0 to 1.5 mL of blood was collected from each specimen [29]. Blood was gently collected from the ventral tail vein by the application of a slight negative pressure and by immobilizing the head and cranial half of the body with the aid of a transferable plastic tube [19]. The venipuncture site was decontaminated using 70% alcohol. Then a 5-mL plastic syringe with 23 gauge needle was inserted at an angle of around 50°–60° between the 5–15 subcaudal scales on the ventral midline. Special attention was given to avoid lymph dilution during blood collection, since it could change the blood biochemical values and lower the blood cell count due to dilution effects [8, 19]. Blood smears were prepared on site immediately following push slide technique. The blood smears were air dried and stained with Leishman’s stain. Three blood smears were prepared per individual. The rest of the collected blood was quickly transferred to BD Vacutainer® for further studies.

### Analysis of hematological and biochemical parameters

Total red blood cell (RBC) and total white blood cells (WBC) counts were manually quantified using Natt-Herrick’s solution and a hemocytometer chamber [30]. Packed cell volume (PCV) was determined using the microhematocrit method. For each snake, three heparinized microhematocrit capillary tubes filled with whole blood were centrifuged for 5 min at 12,000 rpm and then used to calculate the PCV. The hemoglobin concentration was estimated using colorimetric method (Fortress Diagnostics, UK). The mean corpuscular volume (MCV), mean corpuscular hemoglobin (MCH) and mean corpuscular hemoglobin concentration (MCHC) were calculated from the RBC count and PCV [6]. BD Vacutainer® tubes containing the blood samples were centrifuged immediately after blood collection for 120 s at 12,000 × g (TGLM 20 II Centrifuge) for biochemical analysis.

The obtained plasma samples were used to determine (three replicates for per individual) plasma protein, albumin and cholesterol; all of the above contents were measured using standard commercial kits (Fortress Diagnostics, UK). The morphological studies on blood cells were carried out using the Axio Scope.A1 (ZEISS). The sizes of the cells and their nuclei were analyzed with the Zen SP2 Vision program. Morphometric characteristics were taken by measuring 100 RBCs (three slides per snake) and 50 WBCs of each cell type for each individual snake except for basophils.

### Statistical analysis

Mean values and standard deviation (SD) of hematological and plasma biochemistry data were calculated from the software SPSS 17 for Windows. Significant differences between means were determined using an independent sample *t*-test model. Results were considered significant at *p* < 0.05.

## Results

All the studied specimens were adults and in good health condition. Smears from all animals were examined and those that showed pathological changes were excluded from the data analysis. Male and female mean SVL and TOTL were 145.96 ± 14.11 cm and 169.94 ± 17.26 cm respectively. Mean body weight was 1600 ± 320 g for both genders. The female snakes were neither gravid nor postpartum at the time of the research period.

### Blood cell morphology

**Fig. 1:**
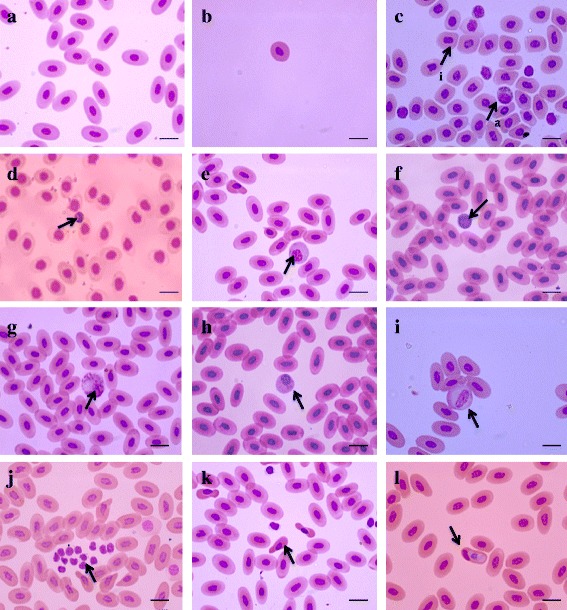
Photomicrographs of peripheral blood cells of *Naja naja* from Sri Lanka. **a** Normal erythrocytes; **b** spheroidal erythrocytes; **c** *a* – Azurophil, *i* – irregular nuclei in red cells; **d** basophiles; **e** large plasmocytoid lymphocytes; **f** small lymphocytes; **g** heterophiles; **h** eosinophils; **i** monocytes; **j** cluster of thrombocytes; **k** poikilocytosis of red cells; **l** *Hepatazon* sp. Horizontal bar: 15 μm

Basophilic (purple) RBC nuclei and eosonophilic (light pink) cytoplasm could be observed under the Leishman’s stain (Fig. 1a). The characteristic morphology of RBC of *N. naja* could be defined as oval or elliptical cells with an oval or even irregular nucleus. However, at times we observed spherical RBC with a spherical or irregular nucleus. The RBC and nucleus measurements (length, width and size) are displayed in Table 1, except for the measurements of irregular shaped RBC or nuclei.

**Table 1:** Characteristics of erythrocytes from *Naja naja* blood in Sri Lanka

Parameters	Male		Female	
	Mean ± SD	Range	Mean ± SD	Range
EL	18.44 ± 0.97	15.94–20.541	17.84 ± 0.88	15.11–18.25
EW	10.69 ± 0.73	10.11–12.01	10.84 ± 0.81	8.1–9.43
ES	159.23 ± 12.22	126.03–187.98	144.32 ± 9.12	114.13–172.34
ENL	7.67 ± 0.92	6.14–9.23	6.71 ± 0.68	5.29–8.65
ENW	5.35 ± 0.47	3.92–5.56	4.76 ± 0.6	3.52–5.02
ENS	33.54 ± 1.82	27.12–38.32	27.23 ± 1.4	21.22–29.23

Average length (EL), width (EW) and surface area (ES) of erythrocytes and nucleus length (ENL), nucleus width (ENW) and surface area of the nuclei (ENS) of erythrocytes in males (*n* = 17) and females (*n* = 13) specimens of *Naja naja* in Sri Lanka. Values are given as the mean ± SD (range)

*SD* standard deviation

The maximum RBC length and width were respectively 20.541 μm and 12.01 μm in all stained blood smears. The male cobras have mean RBC length of 18.44 ± 0.97 μm and a width of 10.69 ± 0.73 μm and mean nucleus length was found to be 7.67 ± 0.92 μm and width was found to be 5.35 ± 0.47 μm. Similarly, female cobras have mean RBC length of 17.84 ± 0.88 μm, width of 10.84 ± 0.81 μm and the mean nucleus length was 6.71 ± 0.68 μm and width was 4.76 ± 0.60 μm. Surface areas of the male RBC (159.23 ± 12.22 μm) was higher in males than female snakes (144.32 ± 9.12 μm).

WBCs of *N. naja* were spherical or oval in shape, categorized into six groups including azurophils (Fig. 1c), basophils (Fig. 1d), large lymphocytes (Fig. 1e), small lymphocytes (Fig. 1f), heterophils (Fig. 1g), eosinophils (Fig. 1h) and monocytes (Fig. 1i). Size characteristics of the WBC types found in *N. naja* blood are given in Table 2. Azurophils are unique to snakes. Lymphocytes and monocytes are agranulocytes while the rest are granulocytes. Eosinophils were occasionally encountered in smears. Eosinophils consisted of granules and nuclei and were either bilobed or concentrated to an end of the cell. These nuclei were thick and slightly notched in the middle. Granules of eosinophils were darkly stained and the cytoplasm was lightly stained.

**Table 2:** Size characteristics of different leukocytes from *Naja naja* blood in Sri Lanka. Values are given as the mean ± SD (range)

Cell Types	Male		Female	
	Mean ± SD	Range	Mean ± SD	Range
Large lymphocytes	14.61 ± 1.99	12.53–18.90	14.01 ± 1.42	12.44–19.88
Small lymphocytes	8.43 ± 1.72	6.32–9.23	9.43 ± 1.72	5.42–10.01
Azurophils	16.01 ± 2.91	10.31–21.23	15.90 ± 2.42	9.32–20.76
Monocytes	14.92 ± 1.91	12.01–17.33	14.01 ± 1.13	12.44–15.88
Eosinophils	10.21 ± 1.1	8.26–11.45	9.44 ± 1.74	7.23–10.11
Basophils	9.81 ± 0.97	6.58–11.32	9.02 ± 0.87	6.00–10.52
Heterophils	18.96 ± 1.89	12.90–21.78	17.88 ± 1.23	15.98–21.00

*SD* standard deviation

Basophils were smaller than eosinophils and contained granules all over the cell, their shape was either spherical or slightly oval. The size of basophils was 9.81 ± 0.97 μm in males and 9.02 ± 0.87 μm in females. Heterophils were large cells with a bi or multilobed eccentric nucleus and coarse cytoplasmic granules. The shape of their nucleus was not clearly visible when the cytoplasmic granules were dispersed within the entire cell. The size varied between individuals and even within the same individual. They resemble snake monocytes in different ways but were differed from azurophils, since azurophils are often smaller and irregular shaped. The nuclei were spherical or oval in shape and contained fine azurophilic granules.

Both small and large lymphocytes were observed on the blood smears. Large (mature) lymphocytes were round in shape or sometimes spherical in shape with abundant cytoplasm compared to smaller lymphocytes. The nuclei were rounded, chromophilic and covered almost the entire cell in smaller lymphocytes. Some of the lymphocytes were plamacytoid in appearance with an eccentric nucleus. We observed that some cells have elongated nucleus. The nucleus was darkly stained while cytoplasm was lighter.

### Hematology and plasma biochemistry

The comparative hematology and biochemical parameters in the blood of *Naja naja* are given in Table 3. The total RBC in male cobras ranged from 0.489 × 10^6^/μL to 0.63 × 10^6^/μL and in female cobras 0.443 × 10^6^/μL to 0.588 × 10^6^/μL. Males showed significantly higher values than females for mature erythrocyte count (*p* = 0.001, t = 8.7627). In addition, females showed significantly lower PCV (*p* = 0.0001, t = 7.0636), Hg (*p* = 0.003, t = 3.0927), MCV (*p* = 0.0483, t = 2.0177) and thrombocytes (*p* = 0.014, t = 2.5327) values. The values of plasma protein, cholesterol and albumin were within the ranges reported for plasma biochemical values of other reptiles. The mean plasma proteins content was significantly different (*p* = 0.0001, t = 9.6692), between males and females; in male cobras were 5.11 ± 0.75 g/mL (2.37 g/mL to 5.97 g/mL) and female cobras it was 3.25 ± 0.74 g/mL (2.19 g/mL to 4.08 g/mL).

**Table 3:** Comparative hematology values (mean ± SD) among the cobras

Species	*Naja naja*		*Naja naja*		*Naja kaouthia*	*Naja siamensis*	*Naja sumatrana*
Sex	M	F	M	F			
References	Current study (Sri Lanka)		Parida et al. [33] (India)		Salakij et al. [26] (Thailand)		
PCV (%)	23.80 ± 2.03^a^	20.41 ± 1.67^a^	29.8 ± 4.2^a^	25.4 ± 3.9^a^	21.2 ± 1.2^a^	21.3 ± 1.8^a^	18.8 ± 2.4^a^
Hemoglobin (g/dL)	7.6 ± 0.89^a^	6.62 ± 1.49^a^	6.9 ± 0.1^a^	6.5 ± 0.2	6.5 ± 0.4^a^	6.9 ± 0.6^a^	4.8 ± 0.7^a^
RBC (10^6^/μL)	0.581 ± 0.035^a^	0.495 ± 0.0408^a^	0.39 ± 0.125^a^	0.3475 ± 0.750^a^	0.616 ± 0.052^a^	0.576 ± 0.042	0.657 ± 0.086^a^
MCV (fL)	397.71 ± 36.51^a^	380.30 ± 30.01	386.7 ± 63.8	348.2 ± 59.33^a^	362.7 ± 18.9^a^	371.6 ± 24.9^a^	289.3 ± 9.6^a^
MCH (pg)	135.43 ± 19.37^a^	141.11 ± 21.96^b^	118.6 ± 23.1^a^	142.4 ± 19.5	110.1 ± 5.90^ab^	120.3 ± 9.62^ab^	71.2 ± 3.1^ab^
MCHC (g/dL)	31.30 ± 14.61^a^	33.42 ± 9.67	38.8 ± 1.9^a^	42.6 ± 1.4^a^	30.5 ± 0.7	32.3 ± 1.5	24.8 ± 1.7^a^
WBC (10^3^/μL)	12.45 ± 1.32^a^	11.98 ± 1.62^b^	11.70 ± 1.00	12.10 ± 2.00	14.316 ± 1.265	12.025 ± 0.880	9.816 ± 1.046^ab^
➢ Azurophils (%)	30.07 ± 3.12^a^	27.88 ± 2.1^a^	~	~	26.1 ± 3.7^a^	25.2 ± 3.5^a^	26.0 ± 4.5^a^
➢ Heterophils (%)	10.05 ± 2.41^a^	8.87 ± 2.53^b^	25.33 ± 3.51^ab^	23.66 ± 4.5^ab^	4.4 ± 1.0^ab^	1.9 ± 0.5^ab^	4.7 ± 1.3^ab^
➢ Eosinophils (%)	2.94 ± 0.88^a^	2.08 ± 1.12	3.66 ± 1.52	2.66 ± 1.52	1.1 ± 0.08^a^	1.4 ± 0.3^a^	0 ± 0
➢ Basophils (%)	1.02 ± 0.92^a^	0 ± 0	4.66 ± 1.52^a^	5 ± 2^a^	0 ± 0	0 ± 0	0.2 ± 0.2^a^
➢ Lymphocytes (%)	64 ± 4.43^a^	66.26 ± 5.2^b^	49 ± 5.43^ab^	47.26 ± 4.4^ab^	66.9 ± 4.4	71.8 ± 3.5^ab^	69.2 ± 4.1^a^
➢ Monocytes (%)	5.6 ± 2^a^	4.66 ± 1.52^b^	4 ± 2^a^	2.66 ± 1.52^ab^	1.2 ± 0.4^ab^	1.0 ± 0.5^ab^	0 ± 0
Total thrombocytes (10^3^/μL)	5.36 ± 2.23	4.13 ± 1.45	~	~	~	~	~
Plasma protein (g/dl)	5.11 ± 0.75^a^	3.25 ± 0.74^ab^	4.53 ± 0.17^a^	1.24 ± 0.08^ab^	5.56 ± 0.43^b^	6.84 ± 0.63^ab^	9.6 ± 0.4^ab^
Cholesterol (mg/mL of serum)	4.09 ± 0.12	3.78 ± 0.42	3.76 ± 0.06	4.72 ± 0.2	~	~	~
Albumin(g/dl)	2.7 ± 1.1	2.1 ± 1.9	~	~	~	~	~

*PCV* packed cell volume, *RBC* red blood cells, *MCV* mean corpuscular volume, *MCH* mean corpuscular hemoglobin, *MHCH* mean corpuscular hemoglobin concentration, *WBC* white blood cells, *SD* standard deviation

Values are given as the mean ± SD (range), values indicated with the same superscript letters are significantly different at *p* < 0.05

## Discussion

Despite the immense popularity of reptile studies in Sri Lanka, to the best of our knowledge, this is the first detailed study that aims to determine hematological reference range values for a venomous snake species. Numerous studies have been conducted on reptile hematology, plasma biochemistry and blood cell morphology in different regions of the world [11–14, 22, 31–37]. In comparison with the other continents, research work conducted in Indian sub-continent on reptile hematology is far behind.

Our findings demonstrate that the peripheral blood cell of *N. naja* consists of erythrocytes, leukocytes and thrombocytes (Fig. 1j). Plasma of *N. naja* was heterogeneous and is similar to all other vertebrates, containing a variety of different substances in different amounts and some containing trace quantities. Hematological and biochemical values vary depending on factors such as age, gender, life stages and other physiological conditions and reptiles have lower RBC counts than that of mammals and birds, in fact turtles have the lowest [16, 22, 32, 35, 38–41]. Our findings on hematological and morphological characterization of blood cells of healthy *N. naja* could be used as a reference in clinical examinations of the Sri Lankan populations.

The characteristic features of mature RBCs of *N. naja* were either elliptical or oval and this shape is similar to other ectothermic vertebrates. These RBC are nucleated and the centrally spherical or oval shaped nuclei oriented along the long axis of RBC. The shapes of RBCs are morphologically similar to other reptile species, but the size of RBCs have been recorded to vary at intra and interspecies level [11–13, 17, 22, 31–33, 35–37, 42–44]. Comparison of Indian and Sri Lankan populations of *N. naja* RBC size shows larger RBC in *N. naja* inhabiting in Sri Lanka. Even though the results of our study show a slight difference of the RBC size between the two sexes, it was not significant.

Several individuals of *N. naja* with irregular or spherical RBCs carrying irregular or spherical nuclei were observed in the current study. Other studies have also observed the presence of irregular nuclei in RBCs of the snakes of families Elapidae and Viperidae [13, 45]. Occasionally, irregular or round RBCs with oval nuclei may be found, especially in anemic reptiles. This is suggestive of an asynchronous maturation of the cell nucleus and cytoplasm, probably owing to accelerated erythropoiesis.

Polychromatophilic erythrocytes are early erythrocytes in maturation and are larger than mature RBC with rounded nuclei and basophilic scanty cytoplasm. As the RBCs mature, the cells get elongated turning into elliptical shape, pale cytoplasm becomes eosinophilic and nuclei get more condensed. Presence of polychromatic erythrocytes, binucleated RBC and increased mitotic figures indicate physiological response to anemia due to either hemolysis or hemorrhage. The smears examined in our study showed no morphological features to suggest any type of anemia. However, one sample showed intracellular parasites (*Hepatozoon* sp. infection) (Fig. 1l). In addition, cytoplasmic inclusions could also be observed in RBCs. These square or rectangular shaped inclusions were seen in some RBC, which were morphologically similar to hemoglobin crystals that are described in the cytoplasm of iguanas [46]. The inclusion body disease described in Boidae and Pythonide families of snakes show eosinophilic inclusions in tissues, red cells and white cells. Small eosinophilic inclusions or hexagonal crystalline inclusions are seen in viral infections such as those caused by iridoviruses [47]. The cause of pale inclusions seen in our study has to be identified using further tests.

Smear from a single sample showed many dysmorphic nuclei with irregular nuclear coders, nuclear budding and different sizes (Fig. 1c). Such changes could be due to dysplastic erythropoiesis and further studies are required to understand the underlying pathology. That sample was not used to determine the average hematological parameters reported here. Some parasites can cause morphological changes in reptilian blood (e.g. *Plasmodium*, *Toxoplasma*, microfilaria and some bacteria). However, all the smears included in the study showed elliptical shaped RBC with minimal changes in size and shapes. The nuclei were spherical to oval in shape with more uniform appearance. Intracellular inclusions described above were not seen in the samples that were used to take the hematological parameters in this study.

The size of the RBCs found in the current study was in accordance with previous studies [33]. It directly affects the gas transportation function and exchange rate of the peripheral blood stream of the reptiles and reflects the position of a species in the evolutionary scale [34, 48, 49]. A comparison of the surface area of RBCs among reptilian orders shows that the largest cells are found in *Sphenodon punctatus*, followed by turtles and crocodilians. The smallest RBCs are found in the family Lacertidae [16, 45, 47, 50].

The RBC count of both genders for Sri Lankan *N. naja* is higher than the recorded values for Indian animals [33]. Both genders, in Sri Lanka and in India, show significant differences of RBC counts between each other. Although these mean values are significantly lower than those described for other captive cobra species such as monocellate cobra (*Naja kaouthia*, *p* = 0.0084, t = 2.7554) and golden spitting cobra (*Naja sumatrana*, *p* = 0.0008, t = 3.6801) [26]. These differences may be due to the species level, physiological conditions, environment and geographic distribution of the animals.

The WBC cell count in both males and females are within the range reported for other species in the genus *Naja* [26, 27, 33], but is significantly higher than *Naja sumatrana* [26]. The WBCs count may vary either naturally or due to infectious diseases, seasons, temperature and species differences or partly due to gender. There was no significant difference between both sexes in the current study; however, these values were significantly different for the golden spitting cobra [26].

In this study, there were no toxic heterophils observed in the smears. Therefore, we can assume that sampled *N. naja* was in good health conditions without any tissue injuries or diseases [19]. However, heterophilia was seen in one of the smears, which also showed many degranulated cells. This may have been caused due to an inflammatory response or reaction to bacterial infection. Severe toxicity features such as cytoplasmic vacuolations and excessive nuclear lobulations were not seen. This heterophil count may be associated with individual factors such as the microorganisms in the natural environment in which snakes live and the vulnerability of an individual snake to stress or to some other extrinsic or intrinsic factors.

In our study, we observed eosinophils in the peripheral blood stream of *N. naja*. Eosinophils are absent in most of the snake species but have been recorded in several previous studies [26, 33]. Those cells were morphologically similar to that of other species in the genus *Naja* and the percentage of eosinophils was within the range reported for other reptiles [6]. In the present work, males had a higher eosinophil count than females. This difference may have been related to the different physiology of the sexes. According to several studies, the increasing level of eosinophils could be due to parasite infestations [19, 47]. Fluctuations of eosinophil counts might be due to seasonal changes [33]. Further studies must be conducted to understand the difference of physiology between the sexes and their potential adaptive values [8].

The low counts of basophils in our study may indicate an absence of any infections in the examined population during the study period. These cells are small, spherical in shape and their cytoplasm was chromophilic and filled with dark purplish granules. Consequently, these granules will cover the entire nucleus. The percentage of these cells is associated with parasitic or viral infections [6]. The percentage of basophils varies greatly among reptile species [46]. In relation to other reptiles, healthy freshwater turtles (e.g. *Pseudemys rubriventris*) have up to 65% basophils, whereas healthy tortoises and turtles have up to 40% basophils [36, 51].

Out of the six categories of WBCs, lymphocytes were the most abundant cells. The function of lymphocytes in *N. naja* is similar to other reptiles, birds and mammals [51]. The lymphocyte count was significantly different in the Indian population of *N. naja*. These values may vary according to intrinsic, extrinsic factors and due to prolong period of isolations. Some environmental factors such as seasonal variations may directly cause the fluctuations of lymphocyte numbers. Several findings suggest that lymphocyte cell count tend to be low in the winter and high during the summer. In addition, the numbers of lymphocytes are likely to decrease when reptiles are hibernating, after which the lymphocyte concentration increases [32, 52]. Nevertheless, in Sri Lanka, there are no distinct seasonal variations and lymphocyte number might not fluctuate compared to temperate countries.

The azurophils, a type of cell unique to reptiles, were also recorded in our *N. naja* blood samples [46]. These cells were morphologically similar to both monocytes and granulocytes. According to our findings, *N. naja* peripheral blood had a high number of azurophils count compared to other reptiles [6, 27, 29]. Azurophils were the second most common WBC type and their percentages are displayed in Table 3. Comparing to other *Naja* species, the azurophil count showed significantly high variations among Sri Lankan populations of *N. naja.*

Thrombocytes were in clumps and were slightly smaller with distinct cytoplasmic border when compared to lymphocytes in our samples. These were similar to small lymphocytes and sometimes difficult to differentiate from each other morphologically similar cells. Unlike mammalian platelets, these bear a central nucleus. These findings can be useful to identify thrombocytes from lymphocytes. The thrombocytes were distributed randomly or in clumps in the smears; however, high thrombocyte counts with many aggregations were noted in a few smears taken from one sample, which also showed high leukocytosis. This could be a manifestation of an inflammatory response where a similar pattern could be observed in mammalians.

According to our results, the PCV value provides the percentage of whole blood that is composed mainly of erythrocytes. These values are closely similar to previously reported values for *N. naja* in India, but significantly higher than other *Naja* species [26, 27, 33]. However, our findings were within the range reported for other reptiles. Therefore, we can assume that captured wild snakes were healthy throughout our study period because a lower level of PCV usually is suggestive of anemia, vitamin or mineral deficiencies, and higher values of PCV results might be due to either dehydration or polycythemiavera, a condition caused by a malfunctioning of the bone marrow. Further, PCV values may vary with temperature and seasonality in reptiles [53, 54].

Results of the current study indicate that the hemoglobin concentration of *N. naja* in Sri Lanka were lower than those reported by Tosunoğlu et al. [24]; *Bothrops ammodytoides* [10]; *Crotalus durissus terrificus* [9]; *Bothrops jararacussu, Eirenis modestus, Platyceps rubriceps* and *Typhlops vermicularis* [24]. Hemoglobin (Hb) is bringing up the greater share of the solid content of the RBCs. According to Wintrobe [34], the concentration of Hb in RBC is similar in all vertebrates. Hb concentration is directly proportional to PCV, unless considerable numbers of immature cells are present [55]. According Szarski and Czopek [17] and Engbretson and Hutchinson [56] there is no significant difference between the Hb values among male and female cobras. However, results of our study shows that male cobras have a significantly higher percentage of Hb than females, which is in agreement with the findings for males by Parida et al. [33]. Moreover, the difference between Indian and Sri Lankan females are not significant. In some reptile species, females have a higher PCV and Hb value than males [57]. The male MCV value was significantly higher than other *Naja* species including the population of *N. naja* in India and king cobra. Highest MCV values are reported for chelonians and lowest in lizards [30, 47]. When the data from other *Naja* species were compared with our studies, MCH values were lower [26].

The values of plasma protein, cholesterol and albumin were within the ranges reported for plasma biochemical values of other reptiles. The mean plasma protein content was significantly different between males and females. These concentrations of total proteins in male cobra in Sri Lanka are within the range of given values in previous studies for *Trimeresurus arbolaris*, *Bothrops schlegelii* and significantly higher than those values reported for Indian *N. naja* [33, 58]. Therefore, it can be assumed that male cobras have richer plasma protein content than females. However, a *N. naja* caught in the wild presented a lower level of plasma protein, comparing to a captive *Naja* of another species [26, 27]. This may be due the prolonged feeding on high protein diets in captive snakes. Consequently, our results may serve as a tool for evaluation of hypoproteinemia and hyperproteinemia of *N. naja* in Sri Lanka.

## Conclusions

The aforementioned values reveal that hematology and plasma biochemistry ranges vary between the two distinct separate geographic areas and at species level in the same genus. According to plasma biochemistry findings in our study, male and female snakes presented significantly different PCV, Hb, RBC, WBC, azurophils and plasma protein concentrations. In conclusion, our study reveal that the 30 healthy specimens of *Naja naja* from different geographic areas in Sri Lanka show significant difference in several parameters according to the distinct isolated geographic locations and some values such as PCV, Hb, RBC, WBC, azurophils and plasma protein concentration presented significant variance between genders. Although we suggest that the present results can be used as a baseline reference data for evaluation of health status of cobra in Sri Lanka, further studies are required to identify whether other factors – age, life stage, seasonal variations and captivity – statistically affect the hematological parameters. To achieve conservation goals of reptiles in the future of Sri Lanka, the establishment of an advanced hematological database is an essential requirement.
